# Antiretroviral Therapy and Viral Suppression Among Active Duty Service Members with Incident HIV Infection — United States, January 2012–June 2018

**DOI:** 10.15585/mmwr.mm6913a2

**Published:** 2020-04-03

**Authors:** Shauna Stahlman, Shilpa Hakre, Paul T. Scott, Brian K. Agan, Donald Shell, Todd Gleeson, Jason M. Blaylock, Jason F. Okulicz

**Affiliations:** ^1^Armed Forces Health Surveillance Branch, Silver Spring, Maryland; ^2^U.S. Military HIV Research Program, Walter Reed Army Institute of Research, Silver Spring, Maryland; ^3^Henry M. Jackson Foundation for the Advancement of Military Medicine, Inc., Bethesda, Maryland; ^4^Emerging Infectious Diseases Branch, Walter Reed Army Institute of Research, Silver Spring, Maryland; ^5^Infectious Disease Clinical Research Program, Uniformed Services University of the Health Sciences, Bethesda, Maryland; ^6^Office of Health Services Policy and Oversight, Office of the Assistant Secretary of Defense for Health Affairs, Falls Church, Virginia; ^7^Navy Bloodborne Infection Management Center, Bethesda, Maryland; ^8^Walter Reed National Military Medical Center, Bethesda, Maryland; ^9^Infectious Disease Service, San Antonio Military Medical Center, San Antonio, Texas.

Human immunodeficiency virus (HIV) infection is a deployment-limiting medical condition for U.S. armed forces in the Department of Defense (DoD) (*1*). HIV management using contemporary antiretroviral therapy (ART) regimens permits effective suppression of viremia among persons in clinical care. Although service members with HIV infection can remain in military service, treatment outcomes have not been fully described. Data from the Defense Medical Surveillance System (DMSS) were analyzed to estimate ART use and viral suppression among DoD service members with diagnosed HIV infection during January 2012–June 2018 (*2*). Among 1,050 service members newly diagnosed with HIV infection during January 1, 2012–December 31, 2017, 89.4% received ART within 6 months of HIV diagnosis, 95.4% within 12 months, and 98.7% by the end of the surveillance period on June 30, 2018. Analyses determined that, among 793 persons who initiated ART and remained in military service for ≥1 year, 93.8% received continuous ART, 99.0% achieved viral suppression within 1 year after ART initiation, and 96.8% were virally suppressed at receipt of their last viral load test. The DoD model of HIV care demonstrates that service members with HIV infection who remain in care receive timely ART and can achieve both early and sustained viral suppression.

DoD routinely screens its service members for HIV infection to ensure force health protection and to protect the battlefield blood supply (*1*). All active duty service members with HIV infection receive care through the Military Health System and can be retained in service if they can perform their duties. Clinical evaluations are performed by military infectious disease physicians following diagnosis of HIV infection and at least every 6 to 12 months thereafter.

Demographic information, military service personnel records, and laboratory data were extracted from the DMSS, which maintains longitudinal service-related and clinical surveillance data for all personnel throughout their military service. All cases of incident HIV infection occurring among active duty service members during January 1, 2012–December 31, 2017, were identified from surveillance data validated against HIV case lists maintained by each military service. Activated reservists and National Guard members were excluded because DMSS does not record accurate follow-up time for reserve or National Guard members. Pharmacy records for dispensed ART prescriptions were obtained from the DoD Pharmacy Data Transaction Service. This analysis was conducted by the Armed Forces Health Surveillance Branch as part of routine medical surveillance efforts on the health outcomes of service members living with HIV infection. Because the branch was conducting this analysis in its capacity as a public health authority providing medical surveillance support to DoD policymakers, institutional review board approval was not required.

ART initiation was assessed for 1,050 service members with incident HIV infection who remained in service for ≥6 months after diagnosis. ART initiation was defined as dispensation of an initial ART prescription during a specified time frame following diagnosis of HIV infection (within 6 months, within 12 months, or by the end of the study period).

Among 1,050 service members with incident HIV infection, 243 (23.1%) were excluded from analysis of continuous ART and viral suppression because of inadequate follow-up time (206; 19.6%) or incomplete viral load testing (37; 3.5%) and an additional 14 (1.3%) because ART history was missing, leaving 793 (75.5%) service members with incident HIV infection and at least 1 year of follow-up for analysis.* The 243 service members who initiated ART but were not included in additional analysis for continuous ART and viral suppression were similar demographically to the 793 who were included and had no evidence of being immunocompromised (median baseline CD4 count = 513 [interquartile range (IQR) = 386–659] cells/μL). 

Continuous receipt of ART and viral suppression were assessed among the 793 persons who remained in service for at least 1 year after ART initiation and who had documented viral suppression within 6 months of ART initiation or a viral load test 6–12 months after ART initiation. Continuous ART was defined as dispensation of at least a 6 months’ supply of ART within 6 months of initiating ART. Viral suppression was defined as a viral load measurement of <200 copies of HIV RNA per mL within 1 year of ART initiation. Viral suppression was also reported at the last viral load test during follow-up 1 year after ART initiation and at the last viral load test of the surveillance period. In addition, viral suppression was calculated for each year of follow-up after HIV diagnosis, as the percentage of service members whose last viral load test during each year of follow-up was <200 copies of HIV RNA per mL, among service members with at least one viral load test during that follow-up year.

The median interval from diagnosis of HIV infection to the first viral load test indicating viral suppression was also calculated overall and stratified by year of HIV diagnosis. In addition, the overall median interval from HIV diagnosis to the last viral load test in the surveillance period was calculated. Median CD4 counts were calculated at baseline and at the last CD4 test during the surveillance period. SAS statistical software (version 9.4; SAS Institute) was used for all analyses.

Among 1,050 service members with incident HIV infection, 939 (89.4%) initiated ART within 6 months of diagnosis, 1,002 (95.4%) within 12 months, and 1,036 (98.7%) by the end of the surveillance period (Table 1). ART initiation within 6 months of diagnosis was more common among older service members, males, and those in the Air Force (Table 1). Initial ART regimens were anchored by integrase strand transfer inhibitors (63.0%), nonnucleoside reverse transcriptase inhibitors (28.2%), protease inhibitors (6.2%), or other combinations of these agents with or without nucleoside reverse transcriptase inhibitors (2.6%). After exclusion of the 243 service members with inadequate follow-up or viral load testing and the 14 with missing history of ART, among the remaining 793 service members, 744 (93.8%) received continuous ART, and 785 (99.0%) had at least one viral load result indicating viral suppression within 1 year after ART initiation (Table 2). Continuous receipt of ART was more prevalent among older service members, non-Hispanic whites, non-Hispanic blacks, males, officers, and pilot/aircrew personnel, compared with their respective counterparts. A high percentage of viral load suppression within 1 year after ART initiation (>96%) was achieved among all demographic subgroups. A total of 772 (97.4%) service members were virally suppressed at their last viral load test during follow-up 1 year after ART initiation and 768 (96.8%) were virally suppressed at their last viral load test of the surveillance period (Table 2). The percentage of service members with HIV infection who achieved viral suppression ranged from 91.6% of 787 persons in the first year of follow-up to 100% of 15 persons in the seventh year (Table 3). The interval from HIV diagnosis to first viral load test indicating viral suppression ranged from 6.9 months (IQR = 4.9–10.9) in 2012 to 2.9 months (IQR = 2.5–4.3) in 2017 (median = 4.6 months ([IQR = 2.9–7.2]). The median CD4 count at baseline was 486 cells/μL (IQR = 342–625) and 717 (IQR = 565–909) at the last test during the surveillance period.

**TABLE 1:** Service members* who initiated antiretroviral therapy (ART) within 6 months, 12 months, or by the end of the surveillance period after diagnosis of incident human immunodeficiency virus (HIV) infection — U.S. Armed Forces, January 2012–June 2018

Characteristic^†^ (total no.)	Time of ART initiation after HIV diagnosis no. (%)		
	6 mos	12 mos	Ever^§^
**Total (1,050)**	**939 (89.4)**	**1,002 (95.4)**	**1,036 (98.7)**
**Sex**			
Male (1,023)	916 (89.5)	976 (95.4)	1,009 (98.6)
Female (27)	23 (85.2)	26 (96.3)	27 (100.0)
**Age group, yrs**			
<20 (31)	27 (87.1)	29 (93.5)	30 (96.8)
20–29 (744)	659 (88.6)	709 (95.3)	733 (98.5)
30–39 (224)	204 (91.1)	215 (96.0)	222 (99.1)
40–49 (44)	42 (95.5)	42 (95.5)	44 (100.0)
≥50 (7)	7 (100.0)	7 (100.0)	7 (100.0)
**Race/Ethnicity**			
White, non-Hispanic (296)	271 (91.6)	283 (95.6)	293 (99.0)
Black, non-Hispanic (483)	418 (86.5)	459 (95.0)	475 (98.3)
Hispanic (160)	150 (93.8)	155 (96.9)	159 (99.4)
Asian/Pacific Islander (30)	27 (90.0)	30 (100.0)	30 (100.0)
Other/Unknown (81)	73 (90.1)	75 (92.6)	79 (97.5)
**Marital status**			
Married (352)	318 (90.3)	338 (96.0)	349 (99.1)
Single (659)	587 (89.1)	627 (95.1)	648 (98.3)
Other (39)	34 (87.2)	37 (94.9)	39 (100.0)
**Service**			
Army (422)	348 (82.5)	394 (93.4)	414 (98.1)
Navy (345)	322 (93.3)	335 (97.1)	343 (99.4)
Air Force (190)	187 (98.4)	187 (98.4)	188 (98.9)
Marine Corps (93)	82 (88.2)	86 (92.5)	91 (97.8)
**Rank**			
Enlisted (965)	861 (89.2)	920 (95.3)	951 (98.5)
Officer (85)	78 (91.8)	82 (96.5)	85 (100.0)
**Occupation**			
Combat-specific (105)	91 (86.7)	99 (94.3)	103 (98.1)
Motor transport (51)	46 (90.2)	49 (96.1)	50 (98.0)
Pilot/Aircrew (16)	14 (87.5)	14 (87.5)	15 (93.8)
Repair/Engineer (264)	244 (92.4)	256 (97.0)	261 (98.9)
Communications/Intelligence (305)	273 (89.5)	290 (95.1)	300 (98.4)
Health care (127)	111 (87.4)	121 (95.3)	127 (100.0)
Other (182)	160 (87.9)	173 (95.1)	180 (98.9)

* Service members were required to have at least 6 months follow-up time after diagnosis of incident HIV infection.

^†^ All demographic and military characteristics ascertained at the time of incident HIV infection diagnosis.

^§^ By June 30, 2018.

**TABLE 2:** Continuous antiretroviral therapy (ART)*^,†^ and viral suppression within 1 year after ART initiation and at last viral load test during the surveillance period,^§^ among active duty service members in military human immunodeficiency virus (HIV) care^¶^ — U.S. Armed Forces, January 2012–June 2018

Characteristic (total no.)	No. (%)		
	Continuous ART	Viral suppression within 1 year	Viral suppression, last test
**Total (793)**	**744 (93.8)**	**785 (99.0)**	**768 (96.8)**
**Sex**			
Male (771)	728 (94.4)	763 (99.0)	746 (96.8)
Female (22)	16 (72.7)	22 (100.0)	22 (100.0)
**Age group, yrs**			
<20 (23)	22 (95.7)	23 (100.0)	20 (87.0)
20–29 (553)	512 (92.6)	547 (98.9)	534 (96.6)
30–39 (178)	171 (96.1)	176 (98.9)	175 (98.3)
40–49 (35)	35 (100.0)	35 (100.0)	35 (100.0)
≥50 (4)	4 (100.0)	4 (100.0)	4 (100.0)
**Race/Ethnicity**			
White, non-Hispanic (207)	199 (96.1)	206 (99.5)	203 (98.1)
Black, non-Hispanic (370)	355 (95.9)	365 (98.6)	357 (96.5)
Hispanic (137)	118 (86.1)	135 (98.5)	132 (96.4)
Asian/Pacific Islander (23)	19 (82.6)	23 (100.0)	23 (100.0)
Other/Unknown (56)	53 (94.6)	56 (100.0)	53 (94.6)
**Marital status**			
Married (257)	241 (93.8)	256 (99.6)	254 (98.8)
Single (507)	474 (93.5)	501 (98.8)	485 (95.7)
Other (29)	29 (96.6)	28 (96.6)	29 (100.0)
**Service**			
Army (300)	278 (92.7)	295 (98.3)	292 (97.3)
Navy (277)	257 (92.8)	274 (98.9)	266 (96.0)
Air Force (149)	144 (96.6)	149 (100.0)	143 (96.0)
Marine Corps (67)	65 (97.0)	67 (100.0)	67 (100.0)
**Rank**			
Enlisted (724)	675 (93.2)	716 (98.9)	699 (96.5)
Officer (69)	69 (100.0)	69 (100.0)	69 (100.0)
**Occupation**			
Combat-specific (74)	72 (97.3)	73 (98.6)	73 (98.6)
Motor transport (37)	34 (91.9)	36 (97.3)	36 (97.3)
Pilot/Aircrew (11)	11 (100.0)	11 (100.0)	11 (100.0)
Repair/Engineer (213)	202 (94.8)	212 (99.5)	209 (98.1)
Communications/Intelligence (231)	212 (91.8)	227 (98.3)	222 (96.1)
Health care (103)	95 (92.2)	102 (99.0)	99 (96.1)
Other (124)	118 (95.2)	124 (100.0)	118 (95.2)

* Continuous ART was defined as having been dispensed at least 180 days’ supply of ART medications within 6 months of initiating ART.

^†^ Service members were required to have at least 1-year follow-up time after ART initiation. In addition, they must have been virally suppressed within 6 months of ART initiation or have a viral load test on file from 6 to 12 months after ART initiation.

^§^ Viral suppression was defined as having a viral load <200 copies of HIV RNA per mL according to any viral load test that was performed within 1 year after ART initiation.

^¶^ All demographic/military characteristics measured at the time of incident HIV diagnosis.

**TABLE 3:** Viral suppression among active duty service members in military human immunodeficiency virus (HIV) care (N = 793),* by year of follow-up — U.S. Armed Forces, January 1, 2012–June 30, 2018

No. of follow-up^†^ yrs	No. with ≥1 viral load test	No. (%) virally suppressed^§^
1	787^¶^	721 (91.6)
2	727	705 (97.0)
3	511	500 (97.8)
4	315	305 (96.8)
5	182	177 (97.3)
6	78	76 (97.4)
7	15	15 (100.0)

* Service members were required to have at least 1 year of follow-up after ART initiation and to have been virally suppressed within 6 months of ART initiation or have a viral load test on file from 6 to 12 months after ART initiation.

^†^ After diagnosis of HIV infection.

^§^ Last viral load of each follow-up year <200 copies of HIV RNA per mL.

^¶^ No. of persons who had a viral load test within 1 year of HIV diagnosis = 787 of 793.

## Discussion

In 2014, based on surveillance data, CDC indicated that 96% of adults with HIV infection in the United States receiving outpatient medical care self-reported currently taking ART, and 98% reported ever taking ART (*3*). In addition, national data indicate that 81.5% to 85.9% of persons engaged in HIV clinical care during 2016–2017 were virally suppressed at their last test (*4*,*5*). Findings from the current analysis suggest that a high percentage of active duty service members receive ART and achieve viral suppression. The Military Health System permits free universal access for active duty service members throughout all aspects of the HIV care continuum, such as routine testing, specialty care evaluations, laboratory monitoring, and ART. The DoD model of HIV care demonstrates that ART and viral suppression goals can be achieved among a segment of the U.S. population who receive clinical care in a large health care system, despite high mobility and geographic dispersal.

Viral suppression among U.S. service members with HIV infection has increased over time. A study of Air Force service members with HIV infection found that 93% attained viral suppression 1 year after ART initiation during 2006–2011, an increase from 78.6% during 2000–2005 (*6*). The U.S. Military HIV Natural History Study, an observational study of military service members and beneficiaries with HIV infection, determined that viral suppression at 1 year after diagnosis among active duty patients who initiated ART during 2000–2007 was 84%, compared with 64% during 1996–1999 (*7*). Since the 1990s, duration of military service after diagnosis of HIV infection has increased substantially, and the number of AIDS-defining illnesses has decreased (*8*,*9*). The combination of more potent ART with fewer adverse effects and the increased availability of single-tablet regimens have likely contributed to improved outcomes, including the high ART uptake and levels of viral suppression noted in this analysis. In addition, the U.S. military mandates periodic evaluations for service members with HIV infection. DoD and service-specific HIV-related policies stipulate that progressive clinical illness or immune deficiency necessitates duty restrictions and, potentially, a referral for medical evaluation for continued service.^†^^,^^§^^,^^¶^^,^** Cumulatively, these policies likely enhance adherence to ART among service members with HIV infection. Viral suppression also has population-level benefits; a recent CDC study of HIV transmission along the continuum of care in 2016 reported that the Treatment as Prevention^††^ strategy can effectively eliminate secondary sexual transmission of HIV from persons virally suppressed on ART (*10*).

The findings in this report are subject to at least two limitations. First, records of dispensed ART medications were used to estimate ART initiation and continued use; no data on adherence were available. However, viral load determinations following ART dispensation suggest a high level of adherence. Second, DoD service members constitute an open population with varying entry and exit dates; therefore, rates of ART use and viral suppression could only be assessed for persons who remained in service during specified periods.

DoD embodies a contemporary national model of successful HIV care, given the high uptake of HIV treatment and achievement of viral suppression by its service members. DoD will continue to review its policies and the scientific literature and report findings of health outcomes among service members living with HIV infection.

**Summary**

**What is already known about this topic?**

U.S. Department of Defense (DoD) service members with human immunodeficiency virus (HIV) infection can remain in military service; however, treatment outcomes have not been fully described.

**What is added by this report?**

During January 2012–June 2018, 93.8% of service members with HIV infection who remained in care received continuous antiretroviral therapy (ART). Viral suppression was achieved in 99.0% within 1 year of ART initiation and in 96.8% at the last test during the surveillance period.

**What are the implications for public health practice?**

The DoD model of HIV care demonstrates that the goals of high ART uptake and viral suppression can be achieved and maintained in a large health care system.

## Footnotes

* Chart review determined that among the 14 persons without documentation of ART receipt, four received ART through civilian care, three were “elite controllers” who had spontaneous viral suppression without ART, three refused ART, three started ART after the end of the surveillance period, and one had provider documentation stating “no indication” because of a CD4 count >500 cells/μL.

† https://www.esd.whs.mil/Portals/54/Documents/DD/issuances/dodi/648501p.pdf.

§ https://www.med.navy.mil/sites/nmcphc/Documents/nbimc/648501p.pdf.

¶ https://static.e-publishing.af.mil/production/1/af_sg/publication/afi44-178/afi44-178.pdf.

** https://www.army.mil/e2/downloads/rv7/r2/policydocs/r600_110.pdf.

†† https://www.hiv.gov/tasp.
